# Supplementary material for: HIVprotI: an integrated web based platform for prediction and design of HIV proteins inhibitors
Source: J Cheminform. 2018 Mar 9;10:12. doi: 10.1186/s13321-018-0266-y (PMC5845081; doi:10.1186/s13321-018-0266-y)
Supplement: Supplementary file 2 — Additional file 2. Source code of HIVProtI web server. [file 13321_2018_266_MOESM2_ESM.zip › HIVprotI_Source-code/hivprotI/weka-3-6-6/doc/weka/classifiers/trees/j48/PruneableClassifierTree.html]

PruneableClassifierTree


---


|  |  |  |
| --- | --- | --- |
| **Overview** \| **Package** \| **Class** \| **Tree** \| **Deprecated** \| **Index** \| **Help** \| **Weka's home** | |  |
| **PREV CLASS**   **NEXT CLASS** | **FRAMES**    **NO FRAMES**     **All Classes** |  |
| SUMMARY: NESTED \| FIELD \| CONSTR \| METHOD | DETAIL: FIELD \| CONSTR \| METHOD |  |


---


## weka.classifiers.trees.j48 Class PruneableClassifierTree

```
java.lang.Object
  weka.classifiers.trees.j48.ClassifierTree
      weka.classifiers.trees.j48.PruneableClassifierTree
```

**All Implemented Interfaces:**: java.io.Serializable, CapabilitiesHandler, Drawable, RevisionHandler

---

``` public class PruneableClassifierTree extends ClassifierTree ```

Class for handling a tree structure that can
be pruned using a pruning set.

**Version:**
:   $Revision: 5535 $

**Author:**
:   Eibe Frank (eibe@cs.waikato.ac.nz)

**See Also:**: Serialized Form

---

**Field Summary**

**Fields inherited from interface weka.core.Drawable**

`BayesNet, Newick, NOT_DRAWABLE, TREE`


**Constructor Summary**

`PruneableClassifierTree(ModelSelection toSelectLocModel,
boolean pruneTree,
int num,
boolean cleanup,
int seed)`
  
          Constructor for pruneable tree structure.


| **Method Summary** | |
| --- | --- |
| `void` | `buildClassifier(Instances data)`             Method for building a pruneable classifier tree. |
| `Capabilities` | `getCapabilities()`             Returns default capabilities of the classifier tree. |
| `java.lang.String` | `getRevision()`             Returns the revision string. |
| `void` | `prune()`             Prunes a tree. |

**Methods inherited from class weka.classifiers.trees.j48.ClassifierTree**

`assignIDs, buildTree, buildTree, classifyInstance, cleanup, distributionForInstance, graph, graphType, numLeaves, numNodes, prefix, toSource, toString`

**Methods inherited from class java.lang.Object**

`equals, getClass, hashCode, notify, notifyAll, wait, wait, wait`

**Constructor Detail**

### PruneableClassifierTree

```
public PruneableClassifierTree(ModelSelection toSelectLocModel,
                               boolean pruneTree,
                               int num,
                               boolean cleanup,
                               int seed)
                        throws java.lang.Exception
```

:   Constructor for pruneable tree structure. Stores reference
    to associated training data at each node.

    **Parameters:**: `toSelectLocModel` - selection method for local splitting model: `pruneTree` - true if the tree is to be pruned: `num` - number of subsets of equal size: `cleanup` -: `seed` - the seed value to use **Throws:**: `java.lang.Exception` - if something goes wrong


**Method Detail**

### getCapabilities

```
public Capabilities getCapabilities()
```

:   Returns default capabilities of the classifier tree.

    :   **Specified by:**: `getCapabilities` in interface `CapabilitiesHandler` **Overrides:**: `getCapabilities` in class `ClassifierTree`
    :   **Returns:**: the capabilities of this classifier tree **See Also:**: `Capabilities`

---


### buildClassifier

```
public void buildClassifier(Instances data)
                     throws java.lang.Exception
```

:   Method for building a pruneable classifier tree.

    :   **Overrides:**: `buildClassifier` in class `ClassifierTree`
    :   **Parameters:**: `data` - the data to build the tree from **Throws:**: `java.lang.Exception` - if tree can't be built successfully

---


### prune

```
public void prune()
           throws java.lang.Exception
```

:   Prunes a tree.

    :   **Throws:**: `java.lang.Exception` - if tree can't be pruned successfully

---


### getRevision

```
public java.lang.String getRevision()
```

:   Returns the revision string.

    :   **Specified by:**: `getRevision` in interface `RevisionHandler` **Overrides:**: `getRevision` in class `ClassifierTree`
    :   **Returns:**: the revision


---


|  |  |  |
| --- | --- | --- |
| **Overview** \| **Package** \| **Class** \| **Tree** \| **Deprecated** \| **Index** \| **Help** \| **Weka's home** | |  |
| **PREV CLASS**   **NEXT CLASS** | **FRAMES**    **NO FRAMES**     **All Classes** |  |
| SUMMARY: NESTED \| FIELD \| CONSTR \| METHOD | DETAIL: FIELD \| CONSTR \| METHOD |  |


---
